# Microstructured Surface Plasmon Resonance Sensor Based on Inkjet 3D Printing Using Photocurable Resins with Tailored Refractive Index

**DOI:** 10.3390/polym13152518

**Published:** 2021-07-30

**Authors:** Nunzio Cennamo, Lorena Saitta, Claudio Tosto, Francesco Arcadio, Luigi Zeni, Maria Elena Fragalá, Gianluca Cicala

**Affiliations:** 1 Department of Engineering, University of Campania Luigi Vanvitelli, Via Roma 29, 81031 Aversa, Italy; nunzio.cennamo@unicampania.it (N.C.); francesco.arcadio@unicampania.it (F.A.); Luigi.ZENI@unicampania.it (L.Z.); 2 Department of Civil Engineering and Architecture, University of Catania, Via S. Sofia 64, 95125 Catania, Italy; lorena.saitta@phd.unict.it (L.S.); claudio.tosto@unict.it (C.T.); 3 Department of Chemical Sciences, University of Catania, Viale Andrea Doria 6, 95125 Catania, Italy; me.fragala@unict.it; 4 INSTM-UDR CT, Viale Andrea Doria 6, 95125 Catania, Italy

**Keywords:** 3D printing, additive manufacturing, photocurable resins, plasmonic, sensor

## Abstract

In this work, a novel approach to realize a plasmonic sensor is presented. The proposed optical sensor device is designed, manufactured, and experimentally tested. Two photo-curable resins are used to 3D print a surface plasmon resonance (SPR) sensor. Both numerical and experimental analyses are presented in the paper. The numerical and experimental results confirm that the 3D printed SPR sensor presents performances, in term of figure of merit (FOM), very similar to other SPR sensors made using plastic optical fibers (POFs). For the 3D printed sensor, the measured FOM is 13.6 versus 13.4 for the SPR-POF configuration. The cost analysis shows that the 3D printed SPR sensor can be manufactured at low cost (∼15 €) that is competitive with traditional sensors. The approach presented here allows to realize an innovative SPR sensor showing low-cost, 3D-printing manufacturing free design and the feasibility to be integrated with other optical devices on the same plastic planar support, thus opening undisclosed future for the optical sensor systems.

## 1. Introduction

In the last 20 years, the progress in the fabrication of novel optoelectronic devices totally flexible, based on organic semiconductor films grown on flexible plastic structures, has been very significant [1,2,3,4,5]. This approach, based on organic semiconductors, could be useful to develop a novel planar technology based on inkjet 3D printing instead of the silicon-based one. The sensing applications, considered the required slow velocities of the components, could be an optimal application field to realize all-plastic sensor systems. In fact, in the last years, novel organic materials (semiconductors, conductors, and insulators) have been developed for the industrial production of devices on large-area, low-cost, plastic substrates [1]. Therefore, a great progress has been made in the field of electronic and optoelectronic devices, e.g., Organic Light-Emitting Diodes (OLEDs) [2], Organic Field Effect Transistors (OFETs) [3], organic sensors and actuators [4]. These plastic devices, totally flexible, will develop in the future because they are simple and cheap to produce (clean Rooms of the microelectronics industry are not necessary). Moreover, works on all-polymer solar cells have been also presented [5].

Polymer optical sensors have been also presented to detect specific substances, such as those based on polymer optical fibers (POFs) and molecularly imprinted polymers (MIPs) [6]. The use of plasmonic surfaces or materials, relying on the surface plasmon resonance (SPR) phenomenon, is a very sensitive approach for the determination of the refractive index variations at the interface between a metallic film and a dielectric medium. The SPR method can be exploited for selective identification and concentration measurement of specific substances in water, when the SPR sensing platforms are used combined with specific receptors. Therefore, the substances that can be analyzed exploiting this type of sensors are pollutants, viruses, toxic metals, pesticides, or any other molecules of interest to be detected in aqueous solutions. Usually, these sensors share the common feature of being based on small devices, low-cost equipment, with the possibility of connecting them to the internet for automatic data acquisition and transmission. In the last few years, different kinds of SPR optical fiber sensing platforms have been developed and combined with several specific (bio or chemical) receptors [7,8,9]. All these devices make use of modified optical fibers that limit the design freedom to the use of simple cylindrical fibers with a severe limitation in terms of the geometry of the sensing unit and of the numbers of sensing units that can be built in one device at once.

In a recent review, Lambert et al. [10] outlined the latest development on plasmonic surfaces manufactured using 3D printing as an emerging and challenging technology to produce complex devices. An interesting advancement was demonstrated by Hinman et al. [11] for SPR biosensing by 3D printing equilateral prisms via stereolithography using a commercial photoactive resin. However, to achieve the SPR required performance, polishing of the printed surfaces before gold sputtering was needed. This approach can be a limiting factor for complex shapes with limited access to all the surfaces. Haring et al. [12] printed different shapes, ranging from cylinders to cubes and pyramids, by microextrusion additive manufacturing using laboratory-made resins filled with silver nano-prisms. This approach allowed to tailor the SPR performances and to print graded structures, but it relied on the synthesis of tailor-made nanofillers and resins thus increasing the final costs. A similar approach was investigated to 3D print biocompatible scaffolds with built-in nano-plasmonic sensors by extruding photoactive inks filled with gold nanorods [13]. The use of 3D printing for micro-optofluidic devices has been developed using POFs [14,15,16] and this is an application field that, potentially, might benefit from the development of 3D alternative approaches that envisage the use of optical active materials. Xu et al. [17] reviewed several sensor applications that might benefit from the use of 3D printing technology.

In this work, we have presented the design, the manufacturing, and the numerical and experimental results of a novel surface plasmon resonance sensor, based on an inkjet 3D-printing process. The manufacturing approach proposed here innovates the approach previously presented to manufacture an optical splitter 1 × 2 50:50 for POFs [18]. In the latter paper, the use was limited to exploit optical adhesives as a substitute to POF with no references to the SPR phenomena. In this paper, we expand the use of the optical adhesive combining it with inkjet 3D printing to obtain an SPR sensor. The use of 3D printing opens different application fields because this SPR polymer sensor could be also integrated with other devices to realize all-polymer components for photonic sensors [19].

## 2. Materials and Methods

### 2.1. Materials

For the manufacturing of the SPR two different resins were used: VeroClear RGD810 and NOA88. VeroClear RGD810 is an acrylic liquid photopolymer with a refractive index equal to 1.531 at 650 nm. VeroClear RGD810 is stiff at room temperature with a heat distortion temperature (HDT) of 45–50 ${}^{\circ }$C and a tensile modulus of 2.5 GPa. The formulation is proprietary, and it was developed by Stratasys specifically for PolyJet 3D printing. The safety data sheet (SDS) reports a complex mixture of acrylate monomers and photoactivators. The SPR was 3D printed using VeroClear RGD810 on a 3D printer Stratasys Objet260 Connex1 (Stratasys, Los Angeles, CA, USA). To manufacture the channels of the SPR device FullCure705 was used as a removable support. FullCure705 is a mixture of acrylic liquid photopolymer, polyethylene glycol, propane-1,2-diol and glycerol used in the printing process as break away support that is simply removed by water jetting after printing. VeroClear RGD810 and FullCure705 were both purchased from OVERMACH S.p.A. (Parma, Italy).

The channels of the SPR 3D printed device were filled with and optical transparent adhesive named NOA88 to manufacture the optical wave guide. NOA88 is a low viscosity (250 cps) UV-curing adhesive with a refractive index equal to 1.56 at 589 nm. The refractive index of the NOA88 is the key parameter allowing to obtain a POF because it is higher than the refractive index of the VeroClear RGD810 resin. Having an absorption range ranging from 315 to 395 nm, it was UV-cured by using a universal lamp bulb with UVA emission (365 nm) and irradiating it for 1 h. The SDS for NOA88 reports a composition based on a mixture of a proprietary mercapto-esters blended with triallyl isocyanurate. Norland Optical Adhesive NOA88 was purchased from Edmund Optics LTD (Nether Poppleton York, UK).

### 2.2. Testing Experimental Setup

**Figure 1:**
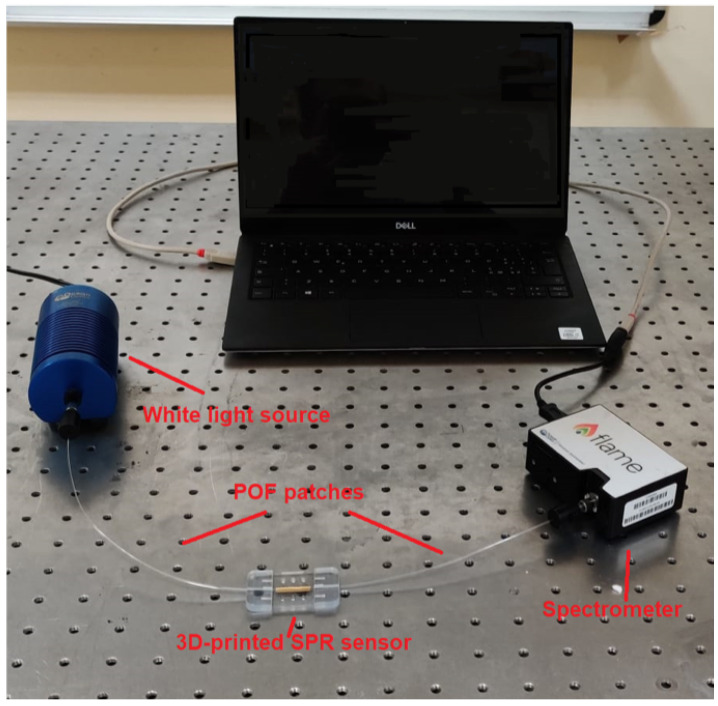
Experimental setup used to test the 3D-printed plasmonic sensor (plastic optical fiber (POF); surface plasmon resonance (SPR)).

To test the developed 3D-printed plasmonic sensor, a very simple experimental setup was adopted, as shown in Figure 1. In particular, a surface plasmon resonance phenomenon has been obtained exploiting a spectral mode configuration (white light source/Spectrometer). This setup includes a white light source (a halogen lamp, model HL-2000-LL, manufactured by Ocean Optics, Dunedin, FL, USA) exhibiting an emission range between 360 nm and 1700 nm; two patches of plastic optical fibers (POFs), both with a total diameter of 1 mm, used to illuminate the 3D-printed SPR sensor and to collect the transmitted light at the output; a spectrometer connected to a Laptop (model FLAME-S-VIS-NIR-ES, manufactured by Ocean Optics, Dunedin, FL, USA), having a detection range from 350 nm to 1023 nm.

### 2.3. Device Design and Process of Fabrication

**Figure 2:**
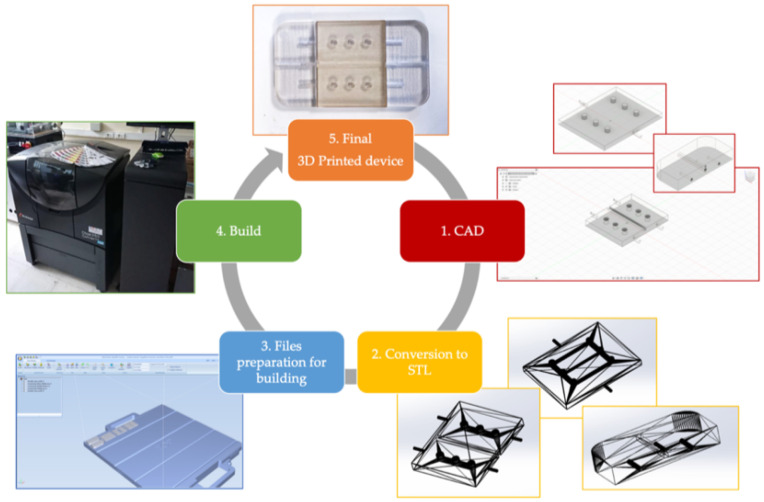
Additive Manufacturing (AM) process steps followed to obtain the physical final device. STL files are provided in the Supplementary Materials.

The device was designed using Autodesk^®^ Fusion 360 and the STL file generated and processed using the proprietary software Objet Studio™ to generate the G-code instructions for the 3D printer. The design and production steps followed to obtain the 3D printed device are summarized in Figure 2. Once the CAD models were designed, they were exported in STL format. Next, each of these files was processed by using Objet Studio™ software with the aim to accomplish the build preparation. Once this step was completed, the build of each part started. This phase was performed by the PolyJet 3D printer Stratasys Objet260 Connex 1. The device was thoroughly created by jetting tiny droplets of liquid photopolymer ink (VeroClear RGD810) onto the build tray, which were instantaneously photocured via the 3D printer’s UV lamps. Near complex geometries, such as holes or overhangs, in addition to VeroClear polymer, were printed using a support material (FullCure705). Once the printing was finished, the support material was washed out with water jetting. Moreover, in order to eliminate support residue and to give a smoother and cleaner surface finish, each part was soaked in a 1% solution of sodium hydroxide, according with the Stratasys post-printing process guide.

**Figure 3:**
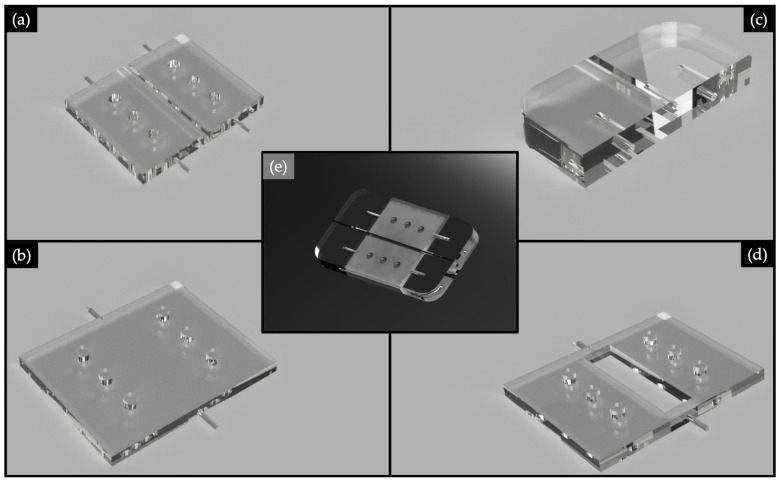
Rendering of the: substrate (**a**), cover (**b**), plastic optical fibers’ (POFs’) support (**c**), mask (**d**) and assembled device (**e**).

The 3D printed device was designed as an assembly of four different parts. In Figure 3 is represented a rendering of each part and of the device assembly. It was performed by using Autodesk^®^ Fusion 360.

**Figure 4:**
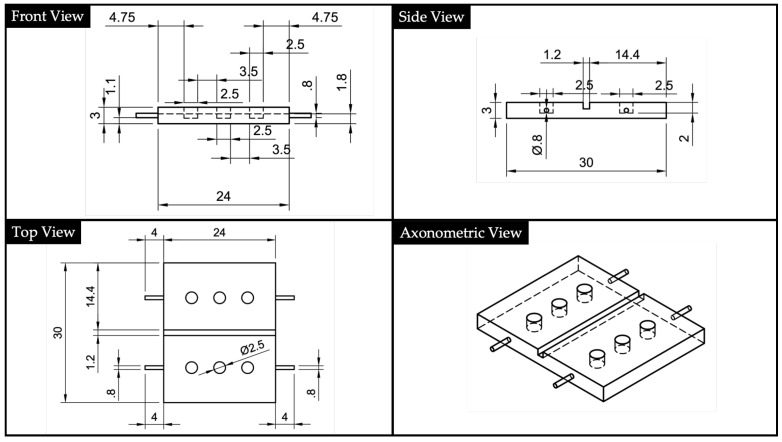
2D drawings and North-West (NW) axonometric view of the bottom part (i.e., substrate).

The geometry of the bottom part, which we will name substrate, is represented in Figure 4. The bottom part was designed with a central square section channel having a dimension of 1.2·1.2 mm${}^{2}$ This cavity hosted the UV-cured optics acting as the waveguide core in the final assembled device. Channel dimension was selected to fit with the POFs used to lead input and output signals. Using this design, the substrate printed using VeroClear RDG810 acted as the cladding for the waveguide core made using NOA88.

**Figure 5:**
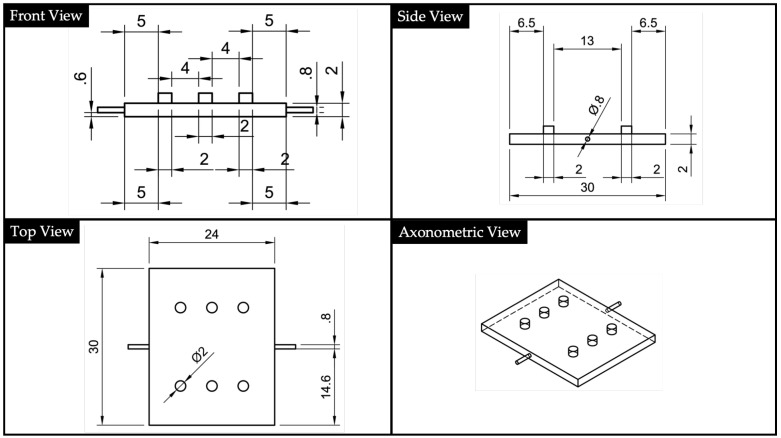
Cover’s 2D drawings and North-West (NW) axonometric view, obtained via Autodesk^®^ Fusion 360 and according with the ISO standard.

Based on the geometry of the substrate, a cover was designed with the aim of cladding the uncovered upper part of the core. Its geometry is reported in Figure 5. To improve the fitting between the substrate and the cover, suitable holes (for the substrate) and centering pins (for the cover) were designed allowing the centering of the two parts and avoiding the presence of an air layer.

**Figure 6:**
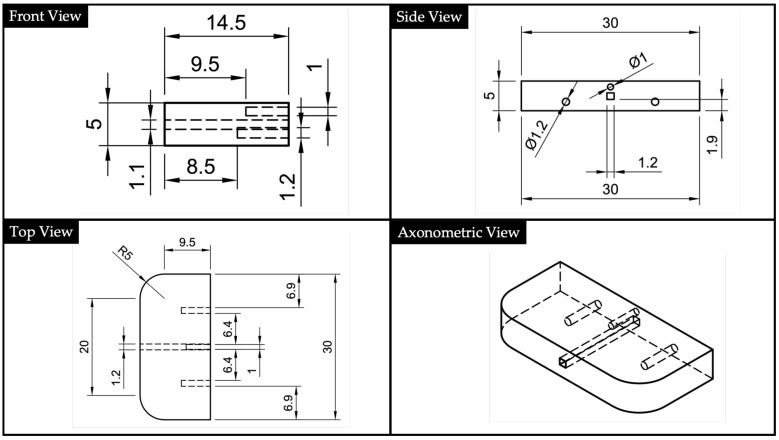
Plastic optical fibers’ (POFs) supports’ 2D drawings and North-West (NW) axonometric view, obtained via Autodesk^®^ Fusion 360 and according with the ISO standard.

To ensure an appropriate insertion of the 1 mm POF waveguides, two supports were designed (Figure 6). The supports have square section channels with a dimension of 1.2·1.2 mm${}^{2}$, and they also have three holes to be coupled with the centering pins manufactured in the substrate and the cover. In fact, looking at Figure 6 it is possible to notice that laterally both have centering pins. The rationale to split the device’s body from the input and output ports is to avoid leakage of the liquid UV-curing adhesives into the channels used for POFs insertion. Channels with polymeric adhesive smears could negatively interfere with both the input light signal and acquired one at the output. Therefore, with our assembled manufacturing process, we avoid the presence of interface surfaces that can impair the light transmission.

**Figure 7:**
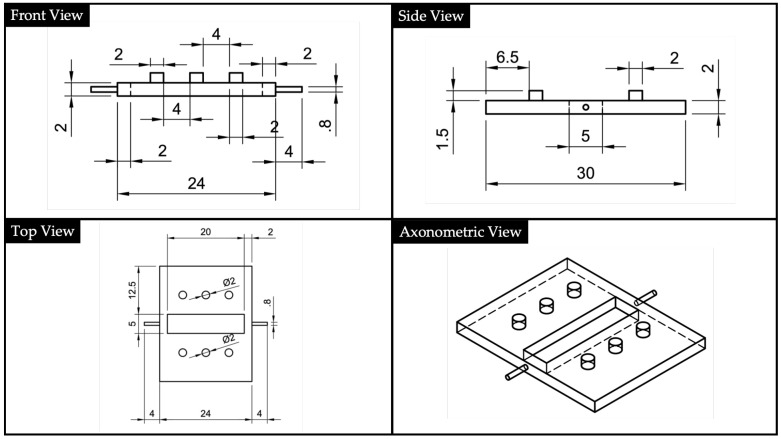
Mask’s 2D drawings and North-West (NW) axonometric view, obtained via Autodesk^®^ Fusion 360 and according with the ISO standard.

To trigger the SPR phenomenon it was necessary to sputter a thin layer of a noble metal (i.e., gold) on the dielectric UV-curing adhesive polymer core. A mask having the appropriate dimensions was designed to gold sputter the core waveguiding only. Mask’s geometry is shown in Figure 7.

**Figure 8:**
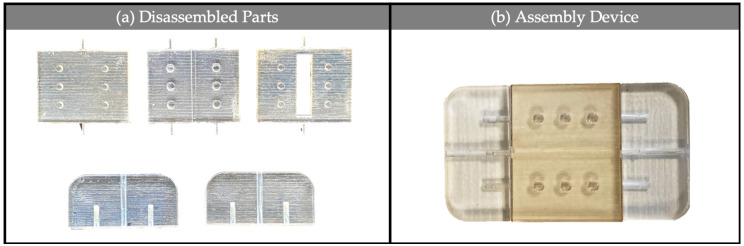
3D printed parts in VeroClear RGD810 (**a**) and assembled device (**b**).

The parts after printing and the assembled device are shown in Figure 8.

**Figure 9:**
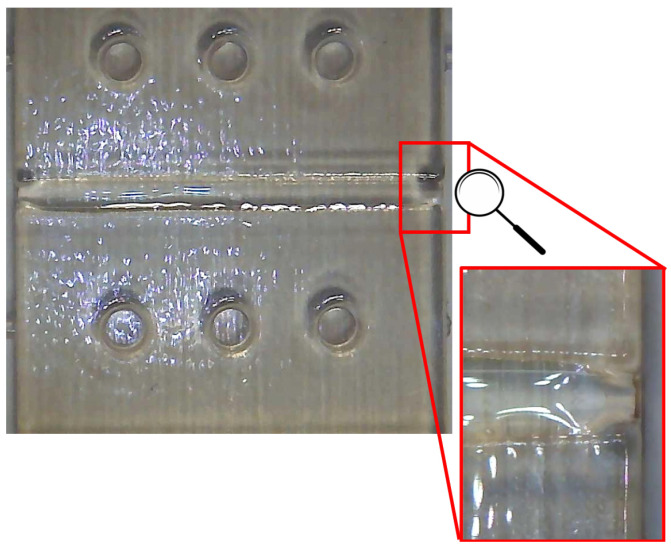
Digital microscope images acquired at variable magnifications (ranging from 50× to 1000×).

The next step was to create the waveguide core of the optical device. Thise step was realized by microinjecting the NOA88 UV photopolymer adhesive into the substrate channel using a syringe with a needle having a diameter equal to 0.5 mm. To avoid spilling outside the canal through the lateral openings, the latter were appropriately occluded using paraffin films. The photopolymer was cured by using a universal lamp bulb with UVA emission (365 nm). The photopolymer was cured by using a universal lamp bulb with UVA emission (365 nm). The photopolymer was irradiated for 10 min. To verify that the NOA88 was injected into the channel correctly, with good adhesion to the channel walls, no micrometric air bubbles and with a mirror surface (to avoid backscattering) in correspondence with the POFs input/output interface areas, the parts were checked using a digital microscope. As shown in Figure 9, no macroscopic defects were identified.

Ultimately, having mounted the designed and printed mask, a thin gold film was deposited by a sputtering process, obtained by a sputter coater machine (model Bal-Tec SCD 500, Schalksmühle, Germany). The thickness of the deposited gold film is about 60 nm. To perform a low-temperature process, the sputtering procedure was iterated for three times (20 nm per step). Each of the three deposition steps was performed for 35 s, at 0.05 mbar of pressure and with a current of 60 mA.

**Figure 10:**
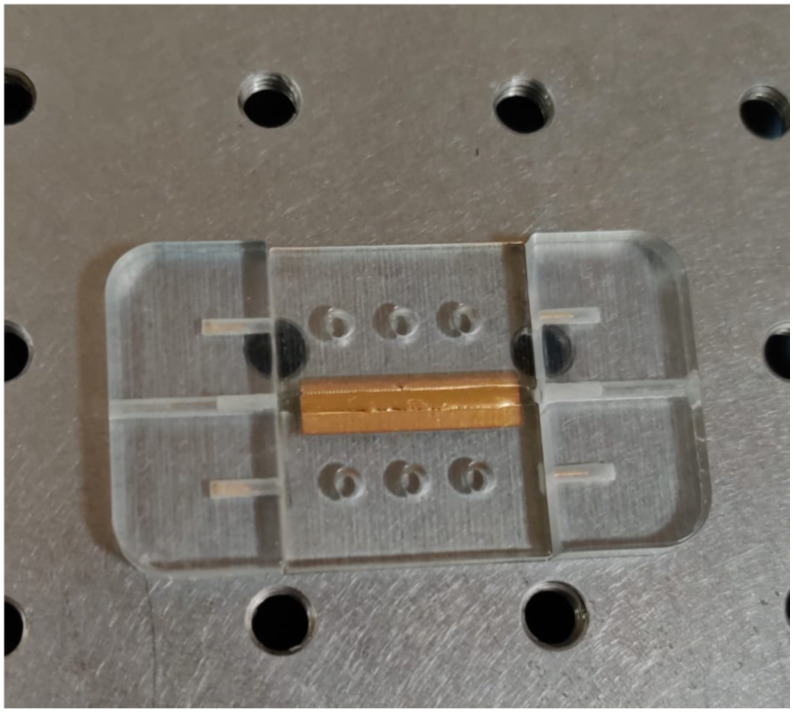
3D-printed surface plasmon resonance (SPR) sensor.

Having designed the mask with a window having a width (equal to 5 mm) larger than the core one, it was possible to avoid the presence of shadow areas during the deposition process, thus guaranteeing a uniform gold coating as shown in Figure 10.

**Figure 11:**
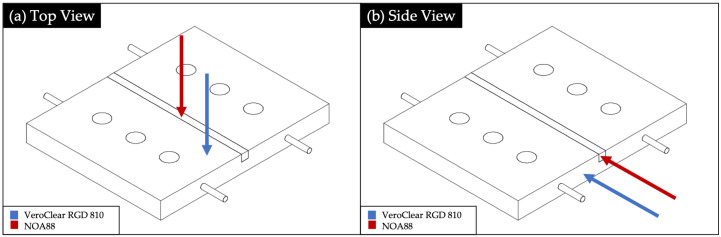
Surfaces of the surface plasmon resonance (SPR) device analyzed by atomic force microscopy (AFM): (**a**) top view; (**b**) side view.

For imaging and measuring the sensor surfaces on a fine scale, the Scanning Probe Microscopy (SPM) approach was used. In particular, the roughness of both the 3D printed device and the NOA88 waveguide was evaluated by mean of the Atomic Force Microscopy (AFM). In AFM, the probe tip is affixed to a cantilever beam. The probe interacts with the surface and the resulting force deflects the beam in a repulsive manner, as described by Hooke’s Law. In the same manner that a spring changes dimensions under the influence of forces, the attractive and repulsive forces between atoms of the probe and the surface can also be monitored when brought extremely close to each other. Hence, the net forces acting on the probe tip deflect the cantilever, and the tip displacement is proportional to the force between the surface and the tip. As the probe tip is scanned across the surface, a laser beam reflects off the cantilever. By monitoring the net (x, y, and z) deflection of the cantilever, a three-dimensional image of the surface is constructed [20]. The AFM measurements were carried out by mean of an AFM NTEGRA by NT-MDT. The test was run in a semicontact mode, with a rate equal to 0.5 Hz, and by using a tip HA-NC (ETALON) characterized by a resonant frequency of 140 ± 10% kHz. The measurements were carried out both in the front view (i.e., in the surface though which the light source enters the waveguide), and in the upper view (i.e., the interface between the NOA88 waveguide and the gold film) of the device (Figure 11).

## 3. Results

### 3.1. AFM Analysis

The analysis of these two mentioned areas is quite important, since an accentuated roughness in the input zone for the light source may cause scattering phenomena, by altering the device functioning. Conversely, the top surface quality is important in the interaction with the gold layer in order to trigger the SPR phenomena. Both the 3D printed part and the photopolymer adhesive of each mentioned view (Figure 11) were investigated, thus, to compare the roughness of each material used. The AFM analysis were run in different locations, but in each of them a 5 × 5 μm^2^ area was investigated. Eventually, for each analysis conducted three parameters were evaluated: the roughness distribution in the square surface (RMS), average roughness (RA), and the Peak to Peak parameter.

**Figure 12:**
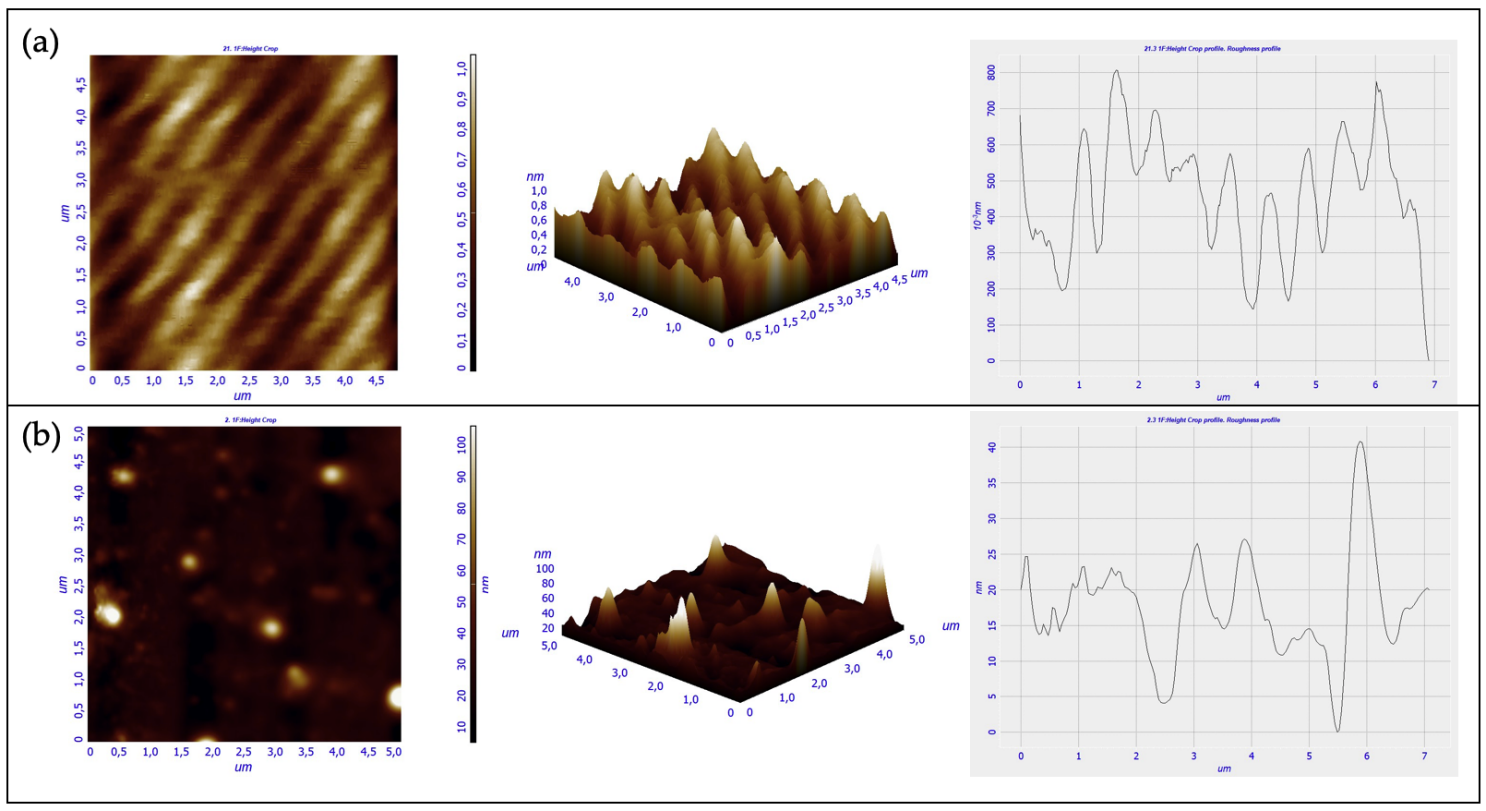
Measurements conducted from the top view in both VeroClear RGD 810 (**a**) and NOA88 (**b**).

**Figure 13:**
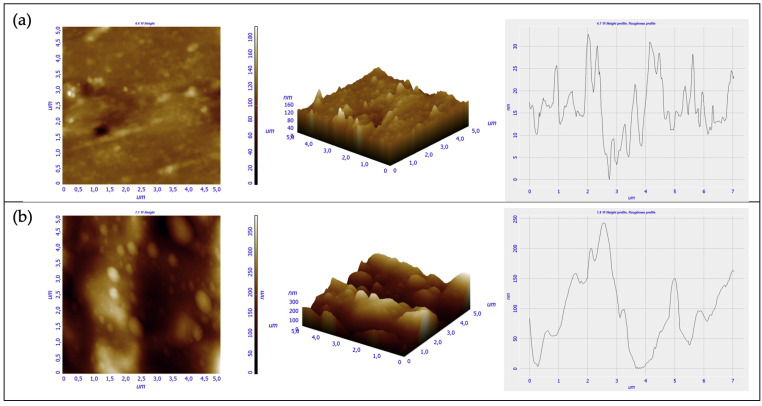
Measurements conducted from the side view in both VeroClear RGD 810 (**a**) and NOA88 (**b**).

While the obtained results from the top view analysis are shown in Figure 12, in Figure 13 are illustrated the ones related to the section analysis to investigate the side view.

The surface roughness of the side view is higher than the top one (Table 1) for both the materials used. The result is justified by the technologies used to manufacture the device and the waveguide. The inkjet 3D printing is a layer by layer manufacturing technique with layer thickness depending on the complex combination of several parameters like: droplet size, droplet splaying, resin shrinkage, platform movement etc. The surface on the side view is rougher than that of the top view, because the layers were exposed in this view. The surface’s roughness measured for the waveguide was related to the manufacturing approach used because, to inject the photopolymer adhesive into the channel, it was necessary to occlude the open ends of the channel. The channel’s open ends were covered by multiple layers of parafilm which were removed once the adhesive was photo-cured. Therefore, the measured roughness of the waveguide was caused by the parafilm closing.

Instead, by focusing on the two different materials used, the results showed that the VeroClear RDG 810 3D printed resin presented a smoother surface than the adhesive NOA88 ones in the two considered view. Eventually, the AFM revealed the presence of small air bubbles, having the dimensions equal about to 350 nm, at the top of the NOA88 waveguide, which by mean of the digital microscope were not identified.

The RMS measure on the different views for the VeroClear RGD810 (0.163 nm and 10.508 nm) and for the NOA88 (12.333 nm and 70.862 nm) are smaller than the values (i.e., 326 nm) reported for optical components obtained by stereolithography previously [11] confirming that the approach followed yielded high quality devices. However, the highest RMS was measured for the side view of the waveguide (i.e., 70.862 nm) where POFs for light input and ouput are passing. The presence of this roughness can create some dispersion and backscattering effects that could impact on the device’s performances.

### 3.2. Numerical Results

To develop the plasmonic sensor in polymer waveguides, in a first step, a numerical study was conducted to predict the optical response of the conceived SPR sensor.

**Figure 14:**
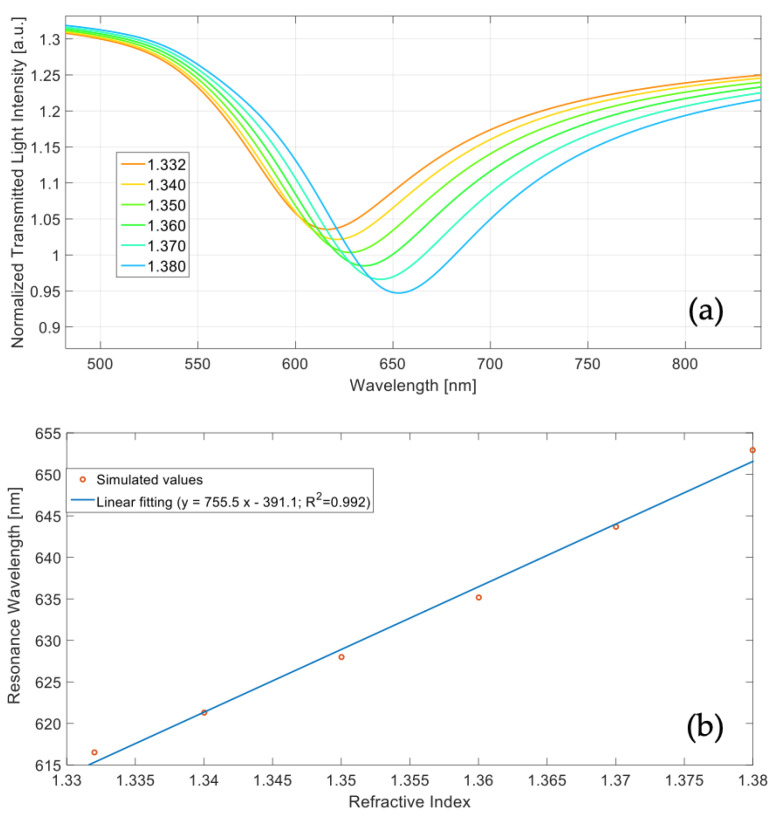
(**a**) Simulated surface plasmon resonance (SPR) transmitted spectra at varying of the external refractive index. (**b**) Resonance wavelength versus refractive index and linear fitting of the simulated values.

This numerical approach was based on an N-layer approximation and takes advantage of the transfer matrix formalism reported in [21]. Figure 14a shows the simulated SPR transmitted spectra at varying of the external refractive index (from 1.332 to 1.380), whereas Figure 14b reports the resonance wavelength as a function of the refractive index, together with the linear fitting of the simulated values.

The SPR spectra reported in Figure 14a were obtained by normalizing the transmitted spectra with the reference spectrum, achieved when considering air as surrounding medium (*n* = 1); in fact, in air the SPR condition was not satisfied.

The performances of these kinds of SPR sensors could be analyzed through the sensitivity (*S*), the signal to noise ratio (*SNR*), and the figure of merit (FOM) parameters. In the spectral mode configuration, at a fixed external refractive index $n_{s}$, these sensors’ parameters can be defined as recalled in the following. If an alteration in the surrounding medium refractive index ($\delta n_{s}$) produces an alteration in the SPR wavelength equal to $\delta \lambda_{res}$, it follows that the sensitivity ($S_{n}$) of the sensor can be defined as [22]:

(1) $$S_{n}=\frac{\delta \lambda_{res}}{\delta n_{s}}\left[nm/RIU\right]$$

From Equation (1) and the linear fitting function reported in Figure 14b, the sensor sensitivity resulted equal to about 755 nm/RIU (the slope of the linear fitting).

In this type of sensors, the *SNR* parameter is related to the easiness in determining the resonance wavelength from the SPR peak and is typically contingent on the width of the SPR curves. So, the *SNR* can be defined as [22]:

(2) $$SNR\left(n_{s}\right)={\left[\frac{\delta \lambda_{res}}{FWHM}\right]}_{n_{s}}$$

where FWHM can be calculated as the Full Width at Half Maximum (FWHM) of the SPR curve. Therefore, the narrower is the SPR curve (i.e., the minor is the FWHM), the greater is the signal to noise ratio.

Lastly, the FOM parameter can be defined as the ratio between the sensitivity and the FWHM, at a fixed external refractive index $n_{s}$, as reported in the following equation [8]:

(3) $$FOM\left(n_{s}\right)={\left[\frac{S}{FWHM}\right]}_{n_{s}}\left[RIU^{-1}\right]$$

### 3.3. Experimental Results

To carry out the experimental measurements, several water-glycerin mixtures were used to change the refractive index value in contact with the gold nano-film. In particular, the refractive index of the solutions (*n*) ranges from 1.332 to 1.382 and these values were previously determined by an Abbe refractometer (Model RMI, Exacta + Optech GmbH, Munich, Germany).

**Figure 15:**
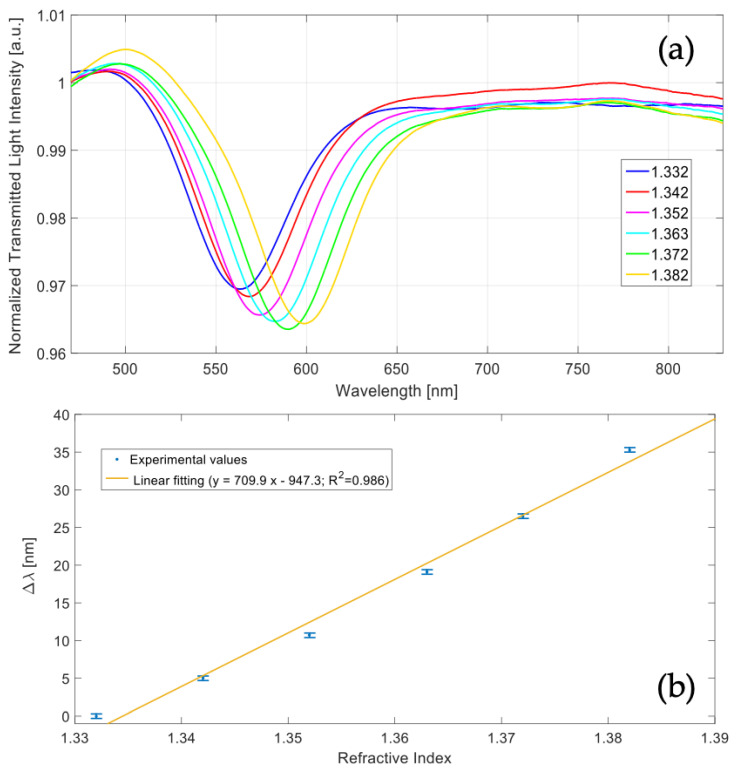
(**a**) Surface plasmon resonance (SPR) transmitted spectra obtained at different refractive indices. (**b**) SPR wavelength variations with respect water (n=1.332) along with linear fitting of the experimental data and error bars.

Figure 15a reports the experimentally measured SPR spectra, obtained by normalizing the transmitted spectra with the one achieved when considering air as the surrounding medium. As it is clear, when the refractive index of the water-glycerin solution increases, the SPR wavelength increases as well.

Figure 15b shows the variations in SPR wavelength (Δλ), calculated with respect to water (n=1.332), along with the linear fitting of the experimental data. In Figure 15b, each experimental value is the average of three different measurements, obtained in a similar condition, and the respective standard deviation is shown as error bar.

## 4. Discussion

As for the numerical study, the sensitivity obtained experimentally can be approximated as the slope of the linear fitting function, reported in Figure 15b , and resulted equal to about 710 nm/RIU in the considered refractive index range. This value is quite similar to the one achieved in the numerical study, showing a good correlation with the theoretical analysis. The sensitivity calculated in the numerical study resulted slightly higher and this is related to the approximation implemented in the theoretical model. In fact, when considering a multilayer-based waveguide (instead of the realized waveguide), a higher number of modes are considered so obtaining both a slightly better sensitivity and broader SPR spectra.

Moreover, the obtained minor value of the Full Width at Half Maximum (FWHM) of the SPR curve with respect to the simulations and other SPR sensor configurations based on multimode POFs [22], can be attributed to the modal filter realized in input and output of the SPR sensor, due to the tapered waveguide region, caused by the manufacturing process, as shown in Figure 9. This modal filter improves the signal-to-noise ratio of the SPR sensor thanks to the filtering of the higher modes [23].

A comparative analysis in term of sensitivity, SNR, and FOM has been reported in Table 2, at a fixed refractive index (n=1.352). The reference SPR sensor is based on a D-shaped POF covered by the same gold nano-film [22].

The 3D printed sensor shows a similar figure of merit (FOM) with respect the reference sensor [22]. The most tangible improvement is clearly connected to a better signal to noise ratio whereas the downside is represented by the reduced sensitivity. However, some improvements could be made by improving the surface roughness investigated by mean of the AFM, by applying simple benchtop polishing procedure [11], and by removing the air bubbles presence by mean of the sonication in the photopolymer adhesive NOA88 before its injection inside the channel.

### Cost Modelling

Finally, to estimate the impact of the proposed sensing approach, cost analysis has been obtained. In particular, the cost for manufacturing the optical-fibre-based surface plasmon resonance device was modeled. The costs parameters were categorized as:

- Material costs;
- Machine costs;
- Process costs.

**Figure 16:**
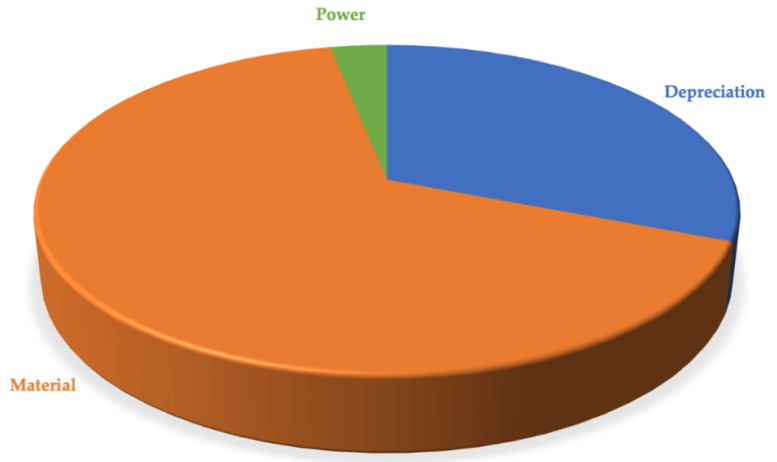
Pie chart relating to the cost allocation of total cost in the cost items.

The material cost was modelled referring to the printing manufacturing that required, for hollow parts, the use of support material (FullCure705). The raw material cost and the quantities of material used for the printing of the whole assembled device, are reported in Table 3. The cost model used considered the depreciation of the machine used (3D printing), since the purchase, installation and maintenance costs of the same are known, the depreciation rate was also considered in the model. The power costs were considered while labour cost was not calculated as the operator is only required to spend a few minutes for print start and part removal from the platform. The power cost calculation considered the power requirements for the instrument used during the fabrication process, by considering as starting point the cost of power expressed in [€/kWh]. Having considered all these aspects discussed so far, the cost allocation was accomplished. The obtained results are shown in Table 4 and Figure 16.

Considering the results obtained from the cost model, it is possible to state that the costs related to energy consumption and depreciation, respectively 3% and 31% of the total, are those that have the least impact on the final price. Vice versa, the costs related to the material (i.e., 66% of the total), have a greater impact on the total cost. However, it must be noted that the high cost of the raw materials used on the Objet printer is justified by the fact that Stratasys uses closed machine with proprietary materials. Recently, some companies developed new printers, based on vat photopolymerization, that allow to use photocurable resins with price ranging at 50 €/kg. The use of such resins would reduce the final cost of the assembled device to about 2–3 €.

## 5. Conclusions

A novel approach to 3D print an SPR sensor was discussed. The sensor was printed using commercial resins and standard inkjet printing. This approach makes the sensor readily available for mass production. The cost analysis resulted in an overall cost of about 15 € which was largely due to the high cost of the photocurable resins. This cost could be further reduced using LCD printing technology that makes use of cheaper resins.

The SPR sensor was CAD modelled and printed. The performances were analyzed using theoretical models for POF based SPR sensor and then experimentally verified. The testing showed a similar figure of merit while a slightly lower sensitivity compared to POF based SPR sensor.

The presented SPR sensor has shown interesting results and its performances could be sufficient to develop a novel kind of plasmonic biochemical sensors for several application fields. For instance, these selective optical fiber sensors can be used for “Smart Cities” applications, as in water quality monitoring, through an IoT (Internet of Thing) approach, or, alternatively, they can be used onboard of simple robots, based on an autonomous guide, to follow increasing concentrations of pollutants in rivers, sea, etc. to identify the point of interest (the source). In all the above applications, weight, cost, and size are very important parameters.

## Figures and Tables

**Table 1:** AFM analysis results.

View Investigate	Material Investigated	RMS [nm]	RA [nm]	Peak to Peak [nm]
Top View	VeroClear RGD810	0.163	0.131	1.094
Side View	VeroClear RGD810	10.508	6.858	192.907
Top View	NOA88	12.333	7.182	140.123
Side View	NOA88	70.862	59.851	388.762

**Table 2:** Performances’ parameters relative to two different types of SPR sensors at a fixed refractive index equal to 1.352.

Sensor	Refractive Index	SNR	Sensitivity ($S_{n}$) [nm/RIU]	FWHM [nm]	FOM [RIU^−1^]	Reference
SPR-POF	1.352	0.982	2.4 · 10 ^3^	181	13.4	[22]
3D-printed SPR	1.352	1.387	710	52	13.6	This work

**Table 3:** Input parameters added to the cost model.

INPUT PARAMETERS		Unit	Value
Material	VeroClear RGD810	€/kg	393.11
	FullCure705	€/kg	126.74
	Norland Optical Adhesive NOA 88	€/mL	2.50
Part	Model	kg	0.017
	Support	kg	0.006
	Printing Time	h	0.47
	Optical Adhesive	mL	1.00
Machine	Depreciation Cost	€/kg	10.00
Process	Power Cost	€/kWh	0.10
	Labour	€/h	0

**Table 4:** Cost allocation and total cost evaluation.

Total Cost for one Assembly Device	%	€
Depreciation	31%	4.67
Material	66%	9.94
Power	3%	0.47
Total Cost		15.08

## Data Availability

Not applicable.

## Supplementary Materials

The following are available online at https://www.mdpi.com/article/10.3390/polym13152518/s1. File S1: STL-file of the substrate (STL). File S2: STL-file of the cover (STL). File S3: STL-file of POF support (STL). File S4: STL-file of the mask (STL).
